# Increase of genetic diversity and clonal replacement of epidemic methicillin-resistant *Staphylococcus aureus* strains in South-East Austria

**DOI:** 10.1093/femsle/fnw137

**Published:** 2016-05-25

**Authors:** Gernot Zarfel, Josefa Luxner, Bettina Folli, Eva Leitner, Gebhard Feierl, Clemens Kittinger, Andrea Grisold

**Affiliations:** Institute of Hygiene, Microbiology and Environmental Medicine, Medical University Graz, 8010 Graz, Austria

**Keywords:** MRSA, Austria, *mec*C, PVL, microarray, epidemiology

## Abstract

*Spa*-typing and microarray techniques were used to study epidemiological changes in methicillin-resistant *Staphylococcus aureus* (MRSA) in South-East Austria. The population structure of 327 MRSA isolated between 2002 and 2012 was investigated. MRSA was assigned to 58 different *spa* types and 14 different MLST CC (multilocus sequence type clonal complexes); in particular, between 2007 and 2012, an increasing diversity in MRSA clones could be observed. The most abundant clonal complex was CC5. On the respective SCC*mec* cassettes, the CC5 isolates differed clearly within this decade and CC5/SCC*mec*I, the South German MRSA, predominant in 2002, was replaced by CC5/SCC*mec*II, the Rhine-Hesse MRSA in 2012. Whereas in many European countries MLST CC22-MRSA (EMRSA 15, the Barnim epidemic MRSA) is predominant, this clone occurred in Austria nearly 10 years later than in neighbouring countries. CC45, the Berlin EMRSA, epidemic in Germany, was only sporadically found in South-East Austria. The Irish ST8-MRSA-II represented by *spa*-type t190 was frequently found in 2002 and 2007, but disappeared in 2012. Our results demonstrate clonal replacement of MRSA clones within the last years in Austria. Ongoing surveillance is warranted for detection of changes within the MRSA population.

## Abstract (precis)

Changes in the epidemiology of MRSA clones in Austria.

## INTRODUCTION

Over the last six decades, methicillin-resistant *Staphylococcus aureus* (MRSA) spread over the whole world and has become a global public health threat. Primarily MRSA was restricted to hospitals (hospital-acquired MRSA), where few, multiresistant strains dominated (Grundmann *et al*. 2010; Kinnevey *et al*. 2014; Stryjewski and Corey 2014). For the last three decades, MRSA could also be found outside this setting (community-acquired MRSA, CA-MRSA) (Kock *et al*. 2014). Most of these CA-MRSA strains carry the Panton-Valentine leukocidin (PVL), a virulence factor associated with soft tissue infection or skin infections (Chambers and Deleo 2009; Albrecht *et al*. 2011; Otto 2013; Kock *et al*. 2014). Another development was the occurrence of so-called livestock-associated MRSA (LA-MRSA) linked with mainly pig farming and is therefore found besides the animals, in people with contact to these. Spread from human to human is rarely seen in LA-MRSA, and LA-MRSA seems to have a reduced repertoire of virulence factors. In Austria, the first LA-MRSA was detected in 2004, with increasing numbers up to now (Huijsdens *et al*. 2006; Witte *et al*. 2007; Zarfel *et al*. 2013). While these changes within the MRSA population were observed, during the last 10 years MRSA predominantly belonging to only a few multilocus sequence typing (MLST) clonal complexes (CC) (CC5, CC22, CC8, CC1 or CC398) emerged worldwide, but appeared at different times, replacing other epidemic MRSA clones (Chambers and Deleo 2009; Wyllie, Paul and Crook 2011). In a recently described example from Germany, CC22-MRSA (Barnim Epidemic strain, UK-EMRSA15) was first detected in 2001 and increased up to 58.6% of the investigated MRSA isolates in 2010, in contrast to CC5/ST228-MRSA I decreased from nearly 50% in 2000 to 2.3% in 2010 (Albrecht *et al*. 2011). Further examples of a replacement of MRSA clones were reported from many countries all over the world, such as Ireland, UK, Portugal or the USA (Enright *et al*. 2002; Amorim *et al*. 2007; Chambers and Deleo 2009; Grundmann *et al*. 2010).

There are a few publications on MRSA epidemiology from Austria, but they mainly focused on CA-MRSA or LA-MRSA (Ruppitsch *et al*. 2006; Krziwanek *et al*. 2007, 2008; Grisold *et al*. 2009).

This study is the first study for Austria, investigating the genetic background of all detected MRSA primary isolates from 2002 up to 2012 in the South-East Austria to determine epidemiological changes. A total of 327 MRSA isolates detected in 2002, 2007 and 2012 in the South-East Austria was *spa* typed, analysed with microarray techniques and assigned to epidemic strains.

## MATERIAL AND METHODS

### Bacterial isolates

The study was performed at the Institute of Hygiene, Microbiology and Environmental Medicine, Medical University Graz, Austria. Clinical samples were obtained from the University Hospital of Graz (approximately 1200 beds), from eight peripheral hospitals and from local practitioners in the district Styria, in the South-East Austria. MRSA isolates of every fifth year were included, resulting in 2629 *Staphylococcus aureus* single-patient isolates in the year 2002, 2823 in the year 2007 and 2772 in the year 2012. From those, MRSA isolates were found in 96 patients (3.65%) in 2002, 72 (2.55%) in 2007 and 159 (5.74%) in 2012. Over study period, only one MRSA isolate per patient was included. MRSA isolates were from hospitalised or from outpatients, distribution was as follows: hospitalised/outpatient was 45 (46.9%)/51 (53.3%) in 2002, 37 (51.4%)/35 (48.6%) in 2007 and 71 (44.6%)/88 (65.4%) in 2012. Distribution to gender was as follows: male/female was 62 (64.6%)/ 34 (35.4%) in 2002, 41 (56.9%)/31 (43.1%) in 2007 and 89 (56.0%)/70 (44.0%) in 2012. Bacterial identification and antibiotic susceptibility testing was performed by using the semi-automated VITEK II instrument (bioMérieux, Marcy l'Etoile, France). Since 2002 all MRSA primary isolates were routinely stored at –70°C. For this study, MRSA were retested, and resistance to cefoxitin was confirmed by Etest (AB Biodisk, Solna, Sweden).

### *Spa* typing

*Spa* typing, DNA purification and PCR were performed as described previously (Grisold *et al*. 2009). The *spa* types and appropriate BURP clusters were assigned by using Ridom StaphType software (http://www.ridom.de/staphtype).

### DNA microarray

For genetic characterization, diagnostic DNA microarray (Identibac, UK; StaphyType, Alere Technologies GmbH, Germany) was used, analysing the MRSA isolates for the presence of over 300 different genes. Protocols have been previously described in detail and are also provided by the kit's manufacturer (Monecke *et al*. 2007).

### Statistical analyses

The statistical analyses were conducted using R^®^ Version 3.2.1, a free software environment for statistical computing (www.r-project.org). Group-specific proportions were tested on their equality by a two-sided binomial test.

## RESULTS

**Figure 1.**
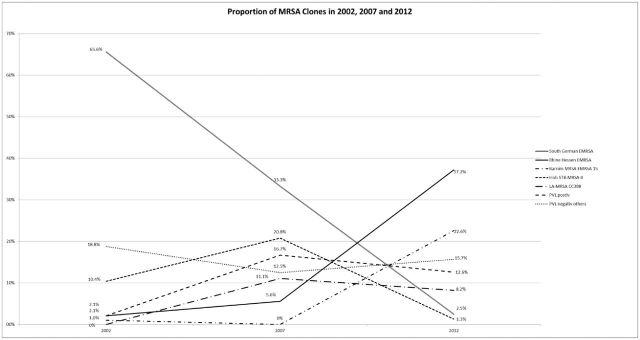
Temporal changes in the MRSA population; PVL-negative MRSA (others) include all MRSA isolates that are not represented in one of the other groups.

A total of 327 MRSA isolates from Austria detected between 2002 and 2012 were investigated, including 96 MRSA isolates from 2002, 72 from 2007 and 159 from 2012. A significant reduction in MRSA cases was observed between 2002 and 2007 (*P* = 0.023). In contrast, a significant increase in the prevalence of MRSA was observed between 2007 and 2010 (*P* < 0.01). MRSA isolates were *spa* typed and analysed with microarray chip. All together, the analysed MRSA isolates belonged to 58 different *spa* types and 14 different MLST clonal complexes (CCs). Concerning *spa* types and MLST CCs in 2002, 14 different *spa* types and six MLST CCs were detected. Eight of these *spa* types were found in 2002 only. In 2007, 20 *spa* types and eight MLST CCs were detected, seven of these *spa* types were found in 2007 only. In 2012, 40 *spa* types and 13 MLST CCs were detected. Notably 28 of these *spa* types and five MLST CCs could be detected in 2012 only. The proportion of the different MRSA clones and the temporal changes are shown in Fig. 1.

### MLST CC5-MRSA

In all three investigated years (2002, 2007 and 2012), MLST CC5 was the dominant clonal complex with 160 of 327 investigated MRSA isolates (48.92%), representing 65/96 isolates (67.71%) in 2002, 28/72 (38.89%) in 2007 and 67/159 (42.14%) in 2012. On the respective SCC*mec* cassettes, the CC5 isolates differed clearly within the three years. In 2002, the CC5/SCC*mec*I (South German EMRSA) was predominant with 63 isolates, represented by the *spa* types t001, t041, t12555 and t12898 and in 2007 with 24 isolates, represented by the *spa* types t001, t002, t041 and t1301. In 2012, CC5/SCC*mec*I was found in four isolates (t001, t010, t041). The decline of the South German EMRSA was significant in both observed time periods, with *P* < 0.01 for each period. The CC5/SCC*mec*II (Rhine-Hesse EMRSA) was found in two isolates (t002) in 2002 and four isolates in 2007 (t002, t003, t014). In 2012, CC5/SCC*mec*II was found as the dominant clone, with 58/159 (37.18%) isolates (t002, t003, t014, t626, t10303), representing a significant increase between 2007 and 2012 (*P* < 0.01). In 2012, also three CC5-MRSA with SCC*mec*IV were detected, two of them t002, which were also positive for the PVL toxin (the only PVL-positive CC5 isolates) and one t579 without PVL. In 2012, also two CC5-MRSA isolates harbouring SCC*mec*V were detected, both of which were assigned to *spa-*type t688.

### MLST CC22-MRSA

The MLST CC22/SCC*mec*IV (EMRSA 15, the Barnim epidemic MRSA) was detected, with one exception, in 2012. In total, there were 36 isolates (22.64%) of CC22/SCC*mec*IV (t022, t032, t223, t515, t845, t11769) occurring in 2012 only, which means a significant increase (*P* < 0.01). In 2002, a single isolate could be assigned to CC22/SCC*mec*IV (t005). One CC22 isolate (t032) in 2012 harboured a SCCmecIV and additionally a SCC*mec*V cassette.

### MLST CC8-MRSA

The MLST CC8 was the second most frequent clonal complex with 26/96 isolates (27.08%) in 2002 and 20/72 isolates (27.78%) in 2007. In 2012, 14/159 isolates (8.80%) were assigned to MLST CC8. SCC*mec* cassettes I-V could be found in isolates from this clonal complex.

The most frequent CC8 clone was CC8/SCC*mec*II with 10 isolates (Irish ST8-MRSA-II clone) in 2002 and 15 isolates in 2007, represented by *spa*-type t190 (no significant increase). In 2012, no isolate with t190 could be found. CC8/SCC*mec*II was represented by two t334 isolates. CC8/SCC*mec*IV (t008, t024, t036, t064, t622, t10437) was detected in a constant number throughout the investigated three years (ten isolates in 2002, four isolates in 2007 and nine isolates in 2012). Within the CC8/SCC*mec*IV isolates, the PVL toxin (US300 clone) was found in five isolates in 2012 (all t008), one isolate in 2007 (t622), but no PVL-positive CC8/SCC*mec*IV isolate was found in 2002. CC8 harbouring SCC*mec*I was represented by only three isolates in 2002 (t008, t051) and one in 2007 (t2023); CC8 with SCC*mec*III was found in three isolates (t030) in 2002 and three isolates (t037) in 2012. The t037 isolates harboured additionally to the SCC*mec*III and a SCC*mec*V cassette.

### MLST CC1-MRSA

In total, MLST CC1 could be assigned to 11 MRSA isolates. Similar to the CC22-MRSA, MLST CC1 was detected, with one single exception, in 2012. In detail, there were 10 CC1 isolates with SCC*mec*IV (t127, t386, t693, t1784), including the single CC1/SCC*mec*IV isolate (t127) from 2007. One CC1 isolate in 2012 (t127) harboured a SCC*mecV* cassette. This isolate and one CC1/SCC*mec*IV (t1784) isolate were PVL toxin positive.

### MLST CC398-MRSA

There was no CC398- MRSA in 2002. In 2007 and 2012, CC398 (LA-MRSA) was represented by 8/72 (11.11%) and 13/159 (8.17%) isolates, respectively. A total of 20 isolates (t011, t034, t1250, t1451) harboured SCC*mec*V; one isolate (t034) revealed SCC*mec*IV, and all isolates were PVL negative. The proportion of CC398-MRSA remained stable between 2007 and 2012 (*P* = 0.63).

### PVL-positive isolates

Within the study period, the number of the PVL-positive isolates increased, with 2/96 (2.08%) PVL-positive isolates in 2002 and 12/72 (16.67%) in 2007 (significant increase *P* < 0.01). No significant change in the percentage of PVL-positive isolates was found between 2007 and 2012 with 20/159 (12.58%) isolates (*P* = 0.53) in 2012. The dominant clones were the US300 clone CC8/SCC*mec*IV, the European CA-MRSA clone CC80/SCC*mec*IV and the Pacific clone CC30/SCC*mec*IV. The European CA-MRSA clone CC80/SCC*mec*IV (t044, t692, t1339, t4152) was detected in all three years with one isolate in 2002 and six isolates each in 2007 and 2012. The Pacific clone could be assigned to eight isolates (all t019), four isolates each in 2007 and 2012. There were two PVL-positive clonal complexes, MLST CC152 and CC88 which occurred only sporadically. The MLST CC152 was assigned to three isolates (one in 2002 and two in 2007), that was the only MLST type, which in this study did not occur in 2012.

### Sporadic PVL-negative isolates

Six MLST CCs were represented by less than 10 isolates within the study period. CC45 was assigned to six isolates; five CC45 isolates with SCC*mec*IV; one (t004) found in 2002, two (t015) in 2007, two (t026, t371) in 2012 and one CC45 isolate (t331) with SCC*mec*V (2012). Two CC30/SCC*mec*IV (t190, t253) isolates without PVL were found in 2007.

All other sporadic PVL-negative MLST CCs were represented by one or two isolates and were all detected in 2012: two CC361/SCC*mec*V (t315), one CC59/SCC*mec*IV (t216), one CC6/SCC*mec*V (t701) and one isolate CC130/SCC*mec*XI (t4335). This isolate was the only one with SCC*mec*XI, so therefore it did not harbour the *mec*A gene but the *mec*C gene.

## DISCUSSION

Infections caused by MRSA have globally reached epidemic proportions and are well established both in the healthcare setting and in the community (Diederen and Kluytmans 2006; Chambers and Deleo 2009; Stryjewski and Corey 2014). In some countries such as the United States, MRSA is among the most common causes of nosocomial infections (Uhlemann *et al*. 2014). The prevalence rate of MRSA in bloodstream infections (BSIs) in Europe is monitored by the European Antimicrobial Resistance Surveillance Network (EARS-Net). Annual rates of MRSA from BSIs reach from 0.7% for Norway to 64.5% for Romania. For Austria, MRSA in BSI was reported with 9.2% in 2013 (European Centre for Disease Prevention and Control 2010, 2014).

With the occurrence of MRSA strains in different settings, hospitals as well as in outpatients or livestock associated, an increasing number of circulating MRSA clones was observed, the clones obviously in competition with each other (Huijsdens *et al*. 2006; Chambers and Deleo 2009; Otto 2013).

At the beginning of this century, the South German type (CC5) was very common in different parts of Europe, Germany or Croatia; also for Austria, the South German MRSA was the dominant clone in 2002 (Budimir *et al*. 2010). But within the next five years, the South German type disappeared and was replaced mainly by the Rhine-Hesse type; this clone was already common in other parts of Europe such as Great Britain or Ireland (UK-EMRSA-3) (Hookev, Richardson and Cookson 1998; Aucken *et al*. 2002) For Austria, as found in this study, the South German type was still the predominant clone until 2007 but disappeared later, with only four MRSA isolates in 2012. It could be noticed that the South German type was replaced by the Rhine-Hesse type, which was the predominant clone in 2012 with 38.18% of all investigated MRSA isolates (Enright *et al*. 2002; Chambers and Deleo 2009; Albrecht *et al*. 2011).

A similar delay could be observed for EMRSA 15. In many European countries, such as Germany, Ireland or Great Britain, EMRSA 15 is the predominant clone since the middle of the last decade (Amorim *et al*. 2007; Marchese *et al*. 2009; Grundmann *et al*. 2010; Albrecht *et al*. 2011). In Austria, the EMRSA 15 was found in one isolate only until 2012, but was the second most common clone in 2012, with 22.64%.

Beside CC5 and CC22, the CC8-MRSA, including the Irish ST8-MRSA-II and the USA300 clone, was continuously found throughout the study period. The Irish ST8-MRSA-II, represented by *spa*-type t190, was frequently found in 2002 (10.42%) and 2007 (20.83%); this *spa* type could not be found in any isolate in 2012. The high occurrence of *spa*190 in Austria was confirmed by a study from Ruppitsch *et al*. (2006) and Schmid *et al*. (2013), investigating MRSA from other parts of Austria, whereas there is no report of *spa*190 from neighbouring countries such as Germany (Ruppitsch *et al*. 2006; Cookson and HARMONY participants 2008; Albrecht *et al*. 2011; Schmid *et al*. 2013). In contrast CC45, the Berlin EMRSA clone which is epidemic in Germany was only found sporadically in Austria throughout the study period.

Although some other PVL-negative MRSA clones occurred sporadically throughout the study period, e.g. the USA600 (CC45) clone. The highest diversity of PVL-negative MRSA clones was found in 2012, including one CC130 isolate harbouring SCC*mec*XI with the new *mec*C variant, up to date only reported once for Austria. Isolates with SCC*mec*XI are described since only a few years, found both in humans and animals, such as cows (Smith and Cook 2005; Albrecht *et al*. 2011; García-Álvarez *et al.*^2011^; Loncaric *et al*. 2013; Kerschner *et al*. 2014).

CA-MRSA showed high clonal diversity in this study with the majority of the strains belonging to a few predominant clones. The most epidemic clonal type among CA-MRSA in Europe is the European CA-MRSA clone (CC80/SCC*mec*IV), found in at least 11 European countries, as described by Rolo *et al*. For Austria, this clone was described first in 2001 and was the dominant CA-MRSA clone throughout the observed time period in this study (Krziwanek *et al*. 2007; Rolo *et al*. 2012).

The USA300 clone (CC8) is epidemic in the USA, where it became a predominant strain. Additionally to the USA, the USA300 spread all over the world, with reports from Asia, Australia, South America and throughout Europe, but this clone did not become the predominant CA-MRSA in these countries. For Austria, the first report was published in 2003 and USA300 is found up to now constantly, but in annually low numbers (Krziwanek *et al*. 2007; Chambers and Deleo 2009; Grisold *et al*. 2009). Since 2004, LA-MRSA/CC398 is also constantly found, mainly concentrated in two regions in Austria (Upper Austria and South-East Austria) with high density of pig farming (Krziwanek *et al*. 2009; Grisold *et al*. 2010). Eight CC1/ SCC*mec*IV with *spa-*type t127 and PVL toxin negative were detected. This LA-MRSA clone is known for harbouring numerous virulence factors and was first described in Austria in 2008, in that report associated with horses. In other European countries, the MRSA t127 clone was also isolated from pigs and cattle (Cuny *et al*. 2008; Franco *et al*. 2011; Alba *et al*. 2015).

This study investigated for the first time the molecular epidemiology of MRSA isolates in Austria within a 10-year period, but there are some limitations of this study. Although all primary MRSA isolates of a huge region in the South-East Austria were investigated, data from other Austrian regions could not be included. It was also not aim of this study to focus in detail on the clinical background or investigate the outcome of hospital hygiene regimes.

To summarise the diversity within the MRSA, population in South-East Austria increased within the observed period from 2002 to 2012. The results showed that the occurrence of some MRSA clones follows the European trends, but with a delay of five to ten years. CC22-MRSA (the Barnim clone), mostly represented in neighbouring countries such as Austria, did not appear before 2012, which means nearly 10 years later than in Germany. Local specifics like the occurrence of the *spa-*type t190 could be observed. Following the dynamics of MRSA, this study documents that new MRSA clones with new features could emerge at any time highlighting the importance of closely monitoring the epidemiology and genetic background of MRSA.

## FUNDING

This study was supported by (Pfizer reference number ).

## ETHICAL STATEMENT

Isolates were obtained as part of routine diagnostic testing and were analysed anonymously. All data were collected in accordance with the European Parliament and Council decision for the epidemiological surveillance and control of communicable diseases in the European community (The European Parliament and the Council of the European Union. 1998; European Commission. 1999). Ethical approval and informed consent were thus not required. Patients were not physically involved and privacy of patients was provided by coding the tested specimens.
